# Forest Fire Smoke Layers Observed in the Free Troposphere over Portugal with a Multiwavelength Raman Lidar: Optical and Microphysical Properties

**DOI:** 10.1155/2014/421838

**Published:** 2014-07-10

**Authors:** Sérgio Nepomuceno Pereira, Jana Preißler, Juan Luis Guerrero-Rascado, Ana Maria Silva, Frank Wagner

**Affiliations:** ^1^Évora Geophysics Center, Rua Romão Ramalho 59, 7000 Évora, Portugal; ^2^Andalusian Institute for Earth System Research IISTA-CEAMA, University of Granada, Autonomous Government of Andalusia, Avenida del Mediterráneo s/n, 18006 Granada, Spain; ^3^Centre for Climate and Air Pollution Studies (C-CAPS), National University of Ireland Galway, University Road, Galway, Ireland; ^4^Department of Applied Physics, University of Granada, Fuentenueva s/n, 18071 Granada, Spain; ^5^Deutscher Wetterdienst, Meteorologisches Observatorium Hohenpeißenberg, Germany

## Abstract

Vertically resolved optical and microphysical properties of biomass burning aerosols, measured in 2011 with a multiwavelength Raman lidar, are presented. The transportation time, within 1-2 days (or less), pointed towards the presence of relatively fresh smoke particles over the site. Some strong layers aloft were observed with particle backscatter and extinction coefficients (at 355 nm) greater than 5 Mm^−1^ sr^−1^ and close to 300 Mm^−1^, respectively. The particle intensive optical properties showed features different from the ones reported for aged smoke, but rather consistent with fresh smoke. The Ångström exponents were generally high, mainly above 1.4, indicating a dominating accumulation mode. Weak depolarization values, as shown by the small depolarization ratio of 5% or lower, were measured. Furthermore, the lidar ratio presented no clear wavelength dependency. The inversion of the lidar signals provided a set of microphysical properties including particle effective radius below 0.2 *μ*m, which is less than values previously observed for aged smoke particles. Real and imaginary parts of refractive index of about 1.5-1.6 and 0.02*i*, respectively, were derived. The single scattering albedo was in the range between 0.85 and 0.93; these last two quantities indicate the nonnegligible absorbing characteristics of the observed particles.

## 1. Introduction

Forest fires, agricultural burns, and the widespread use of wood as fuel for heating and cooking are major sources of pollution related to biomass since they dominate the production of primary carbonaceous particles (see, e.g., [1–4]). The influence of smoke aerosols in terms of direct and indirect radiative forcing, atmospheric chemistry, and visibility reduction is significant (see, e.g., [5–12]). Also, the impact of smoke aerosols on health [13] and biological processes (e.g., [14]) is documented. Concerning Portugal, an average area close to 150,000 ha was burnt every year, in the period of 2001–2011, particularly during the summer periods [15]. Smoke aerosols from biomass burning are one of the key aerosol types in climate research [16, 17], but their optical and microphysical properties in free tropospheric layers are still insufficiently studied. The presence of smoke at these high altitudes permits its transport over long distances, on a transcontinental scale or, ultimately, around the globe [18–22]. This fact leads to one important issue regarding transforming processes, for example, coagulation, condensation, and gas-to-particle conversion, which might occur during the long range transport of smoke particles, leading to changes in the size distribution [23] and thus in the optical properties. Evidence on particle growth during long range transport and ageing (for timescales of weeks) was presented by, for example, [24–26]. This adds complexity to the characterization of this aerosol type. In that context, Alados-Arboledas et al. [27] reported the first vertically resolved observations of a fresh biomass burning plume in the free troposphere over Spain; the transport time was estimated at 24–36 hours. A single smoke plume from forest fires in the north of Portugal observed over our site was recently studied by Preißler et al. [28], although likely to be mixed with other aerosol types.

Despite the recognition of the importance of free tropospheric aerosol layers in the climate forcing, the determination of their temporally and vertically resolved properties still poses numerous problems. The spatial (3-D) and temporal distribution of aerosols are a crucial input in the global atmospheric models to access the influence of aerosols on climate. The limitations of the observational techniques for characterizing free tropospheric aerosols are one of the difficulties to overcome. On the one hand, satellite and ground-based passive remote sensing devices detect the whole atmospheric column and thus cannot separate particles within the boundary layer from layers aloft, that is, in the free troposphere or stratosphere. On the other hand, using in situ techniques on board aircrafts is costly, and therefore not practical for long term studies. One approach consists in using the space-borne lidar observations (with the Cloud-Aerosol Lidar with Orthogonal Polarization, CALIOP), on board of the satellite CALIPSO (Cloud-Aerosol Lidar and Pathfinder Satellite Observations) [29] (Winker et al., 2010). However, CALIOP only allows retrieving the particle backscatter coefficient (180° scattering coefficient) because it only detects elastically backscattered light. The lidar ratio is defined as the ratio of extinction and backscatter coefficients. In general it depends on the different aerosol properties such as the particle size distribution, shape (both related to the relative humidity), and the refractive index (related to the chemical composition and state of mixture). Numerous studies demonstrated that the extinction-to-backscatter ratio (lidar ratio) is a valuable quantity for aerosol characterization [19, 25, 30, 31]. However when the assumption of the lidar ratio is needed, for the retrieval of the extinction coefficient profile, it becomes a main disadvantage, as it may lead to large uncertainties. On the contrary, Raman lidars enable the independent determination of both the backscatter and extinction coefficients, and thus of the lidar ratio. Usually Raman lidar measurements are restricted to night-time measurements. The knowledge of this quantity is essential to accurately convert the retrieved particle backscatter coefficient profiles, obtained with elastic backscatter lidars, into particle extinction coefficient profiles.

In this work, fresh smoke aerosol from forest fires, detected as several plumes in the free troposphere over Portugal, are studied in terms of optical and microphysical properties, based on multiwavelength Raman lidar measurements. The lidar measurements were carried out between 17 and 19 October 2011, while numerous forest fires were occurring in several regions of the western Iberian Peninsula, both in Portugal and in Spain.

In the following sections, the site is briefly presented as well as the multiwavelength lidar system PAOLI (Portable Aerosol and Cloud Lidar), the inversion algorithm for the retrieval of microphysical properties, and other used methodologies. The results are shown and discussed in Section 3 and a summary is presented in Section 4.

## 2. Measurement Site, Instrumentation, and Methodology

### 2.1. Site Description

Évora (38.5° N, 7.9° W, 300 m above sea level (a.s.l)) is located inland in the south-western region of the Iberian Peninsula. The city (<60000 inhabitants) is the capital of Alentejo, a rural region in Southern Portugal, which covers about one-third of the area of Portugal but has a low population density. The distance to the capital, Lisbon, is about 130 km. The regional landscape consists primarily of soft rolling hills and wide plains. No polluting industries exist in the vicinity of Évora; thus local anthropogenic pollution is caused by traffic and, in winter, domestic fuel burning as well [32] (Pereira et al., 2012). Occasionally, anthropogenic aerosol from Europe, forest fire smoke mainly from the north, and centre of Portugal and from Spain or desert dust from the Sahara are transported to the site [33–39].

### 2.2. Instruments and Methods

The Portable Aerosol and Cloud Lidar (PAOLI) of the Évora Geophysics Center (CGE) is a multiwavelength Raman lidar of the type Polly^XT^ [40] and is operated at CGE on a regular basis since the end of 2009. This lidar system is part of both the European Aerosol Research Lidar Network (EARLINET) [41] and the Spanish and Portuguese Aerosol Lidar Network (SPALINET) [42]. It operates with a Nd:YAG laser which emits primarily at 1064 nm and also at 532 and 355 nm by means of a second and third harmonic generators. Six photomultiplier tubes detect the elastically backscattered photons at the wavelengths 355, 532, and 1064 nm, the inelastically backscattered photons at 387 and 607 nm, corresponding to the Raman-shift on nitrogen molecules of radiation at 355 and 532 nm, and also the cross-polarized component at 532 nm.

The profiles of particle extinction coefficients, *α*(*z*), at 355 and 532 nm, particle backscatter coefficients, *β*(*z*), at 355, 532, and 1064 nm, and the particle linear depolarization ratio, *δ*_*P*_(*z*), at 532 nm, can be obtained from PAOLI measurements. Vertical and temporal resolutions are 30 m and 30 s, respectively. The system's technical details and performance were well described before [39, 40, 43].

A set of lidar measurements which were performed during nighttime between 17 and 19 October 2011 were used in the analysis. Therefore both the particle backscatter and extinction coefficients profiles, at 355 and 532 nm, were obtained by applying the so-called Raman method [44], which is based on the independent measurements at the laser wavelength as well as at the wavelength of the inelastically backscattered light. This Raman signal is only affected by particle extinction and thus is independent from particle backscatter. With this method, no assumption of the lidar ratio is necessary for the calculation of particle extinction and backscatter coefficients. For the retrieval of the particle backscatter profiles at 1064 nm, the Klett method was used [45–47]. Vertical smoothing with sliding window lengths of 500 m for particle backscatter coefficients and of 900 m for particle extinction coefficients was applied to the profiles in order to improve the signal-to-noise ratio, and averaging times of about two hours were applied, except in one case. From the obtained Raman profiles of *α*(*z*) and *β*(*z*) the lidar ratios at 355 and 532 nm, LR_355_ and LR_532_, respectively, were derived. Also, the wavelength dependence of the backscatter and extinction coefficients can be determined, for the wavelength pair 355/532 nm, via the well-known Ångström exponents, å_*β*_ and å_*α*_, respectively. It indicates the proportion of the coarse particles relative to smaller particles.

The optical data retrieved with the lidar (2*α*(*z*) + 3*β*(*z*)) were used as input for the inversion algorithm (e.g., [48, 49]), which is based in the relationship between optical data and microphysical particle properties via the well-known Fredholm integral equations of the first kind. A system of five equations is solved numerically using a regularization method with constraints. Previous findings [48, 50, 51] showed that the combination of backscatter and extinction coefficients allows the retrieval not only of particle size parameters but also the complex refractive index. The output data consists in approximations of particle volume size distributions which are used to compute particle effective radius (*R*_eff_), surface area (*S*), and volume concentrations (*V*) as well as the complex refractive index (CRI) and single scattering albedo (SSA). Details on error analysis are given in [49, 50, 52]. Focus was given to smoke layers observed aloft in the free troposphere where the particle concentrations were more prominent as indicated by enhanced magnitudes of the backscatter and extinction coefficients.

Additional information was provided by an automatic sun tracking photometer (CIMEL CE-318-2) also operating at CGE facilities in the framework of the Aerosol Robotic Network (AERONET) [53]. Spectral aerosol optical depths, *τ*, at eight wavelengths in the range from 340 to 1640 nm, and the respective wavelength dependence information were used.

Moreover, three-day backward trajectories arriving at different heights in the free troposphere were computed with the Hybrid Single Particle Lagrangian Integrated Trajectory Model (HYSPLIT) [54], which allowed estimating the source regions of the particles. The arrival times were chosen to be consistent with the periods of the available lidar measurements. Products of the space-borne instrument MODIS (Moderate Resolution Imaging Spectroradiometer), on board of the Terra and Aqua satellites, were also used in order to obtain information on the forest fires occurring on the Iberia Peninsula as well as on the columnar optical data [55–58].

## 3. Results and Discussion

The vertically resolved characterization of the smoke particles that follows, in terms of their optical and microphysical properties, is based on a set of nighttime measurements available from 17 October 2011 to 19 October 2011 at dawn.

**Figure 1:**
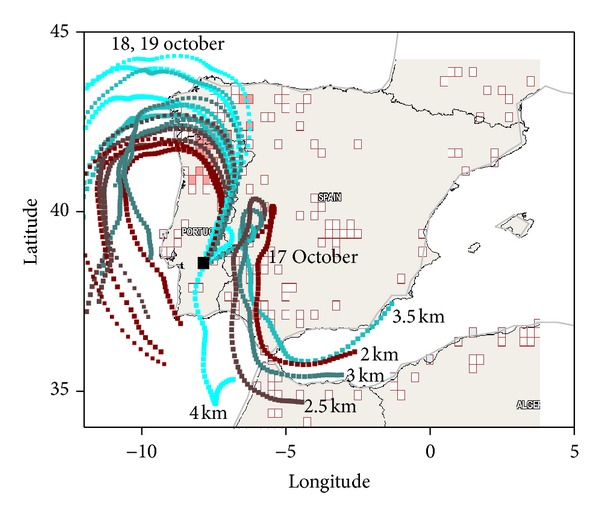
Fire hot spots detected by MODIS on board the Terra and Aqua satellites in the period from 15 to 18 October 2011 (http://firms.modaps.eosdis.nasa.gov/firemap/). 72-hour backward trajectories arriving at Évora (black square) at the lidar measurement times, between 17 and 19 October 2011, at 2, 2.5, 3, 3.5, and 4 km agl. For 18 and 19 October 2011 the colors are the same as for 17 October 2011.

The daytime maximum columnar atmospheric turbidity during this event was recorded by the AERONET sun photometer during the afternoon of 18 October 2011. For that reason, focus was given to lidar measurements performed around this time period, that is, during the previous and following evenings. A large number of forest fires occurred in the preceding days, as shown in Figure 1. The satellite image from MODIS Rapid Response Project indicates a large amount of fires mainly concentrated in the western half of the Iberian Peninsula, particularly in the northwestern areas facing the Atlantic Ocean, as well as in southern Spain. A number of fires were also detected in other Iberian regions as well as in North Africa. Figure 1 depicts the occurrences in the prior days. The same geographical pattern of forest fires occurrences could be observed in a day-to-day basis, as the forest fires were occurring consistently in the same regions. It is worth noticing the absence of fires in the south of Portugal, in a vast region surrounding Évora. Therefore the smoke which was detected at the site that was necessarily transported through distances longer than about 100 km.

The HYSPLIT trajectories drawn in the MODIS fire map were computed for the height range between 2 and 4 km for times centered during the different periods of the lidar measurements. They suggest the transport of particles from the areas with large number of fire hot spots. In the first period of the measurements (evening of 17 October 2011) air masses were transported towards Évora after crossing southern and central regions of Spain. In the subsequent measurement periods (evening of 18 October and 19 October at night) the airflow changed and the trajectories indicated air masses that transported smoke from the forest fires occurring in the northwest regions of the Iberian Peninsula. The backward trajectories suggested a relatively short transport time, between about 1 and 2 days, or even less.

**Figure 2:**
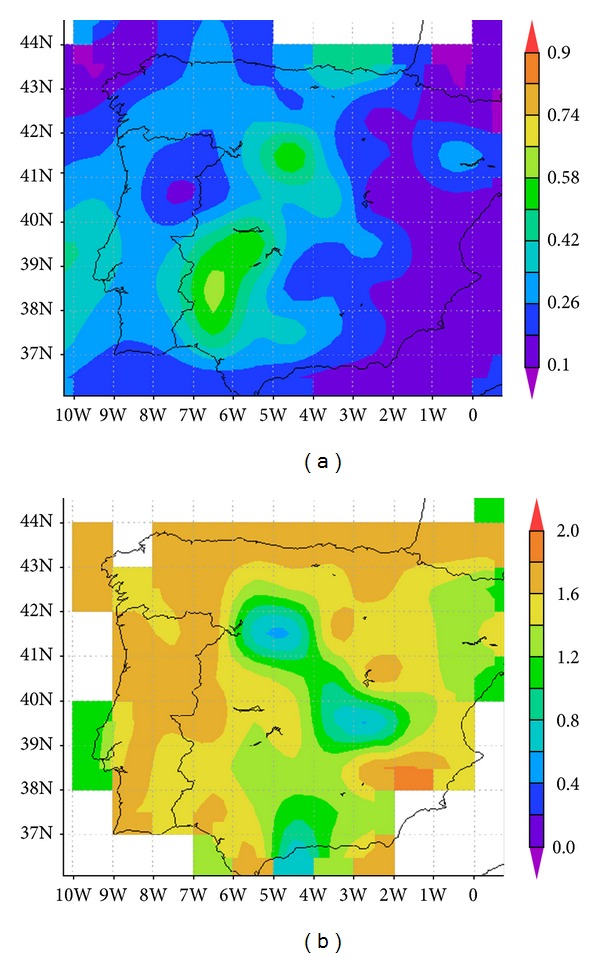
(a) Terra-Modis aerosol optical depth at 550 nm and (b) Terra-Modis Ångström exponent (470/660 nm) on 18 October 2011.

On 18 October 2011 the smoke plumes over Portugal and Spain and the atmospheric turbidity caused by those fires were particularly evident in the MODIS data. The respective Terra-MODIS products for that day, depicted in Figure 2, show high values of both the aerosol optical depth (at 550 nm) and the Ångström exponent (440/670 nm wavelength pair), which was higher than 1.6, over large portions of the Iberian Peninsula, including the region over Évora. The ground-based measurements performed with the sun-photometer are consistent. They revealed aerosol optical depths at 440 nm (*τ*_440_) in the range between 0.39 and a maximum of 0.69, and the average *τ*_440_ for this same day was 0.48 ± 0.07. The corresponding Ångström exponents were consistently high, between 1.5 and 1.7 (at both 440/670 nm and 440/870 nm wavelength pair). They were quite stable during the whole day which is indicated by a coefficient of variation below 5%. This large wavelength dependence indicates that the enhancement in the atmospheric turbidity was due to the presence of small fine mode particles. These records represent a very significant increase in both *τ*, at 440 nm, and å (440/870 nm) when compared to the long term averages of 0.15 and 1.14, respectively, which are characteristic for this site in the long term [59]. Also, they are comparable with other published observations of smoke particles emitted in forest fire events [8, 16, 33, 60].

### 3.1. Aerosol Optical Properties from Lidar Observations

**Figure 3:**
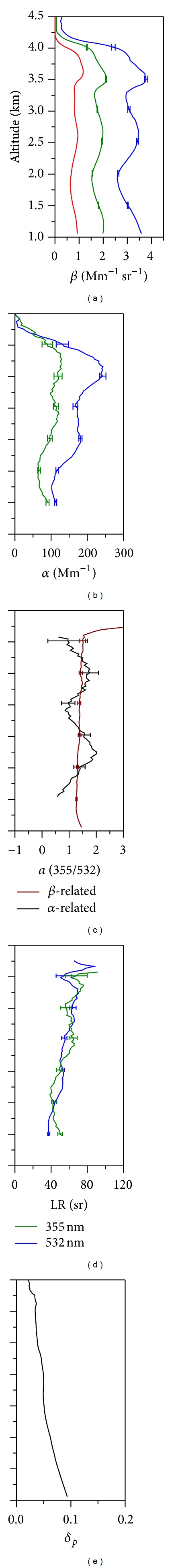
Profiles of (a) backscatter coefficients (355-blue, 532-green, and 1064 nm-red), (b) extinction coefficients (355-blue and 532 nm-green), (c) backscatter and extinction-related Ångström exponent (wavelength pair 355/532 nm), (d) lidar ratios (355 and 532 nm), and (e) particle linear depolarization ratio (532 nm) measured on (a) 17 October 2010 20:20–22:10 UTC.

**Figure 4:**
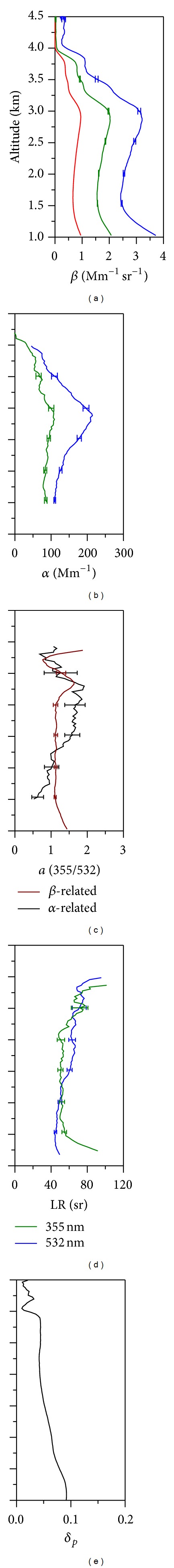
Profiles of (a) backscatter coefficients (355-blue, 532-green, and 1064 nm-red), (b) extinction coefficients (355-blue and 532 nm-green), (c) backscatter and extinction-related Ångström exponent (wavelength pair 355/532 nm), (d) lidar ratios (355 and 532 nm), and (e) particle linear depolarization ratio (532 nm) measured on 18 October 2011 at 19:00–20:45 UTC.

**Figure 5:**
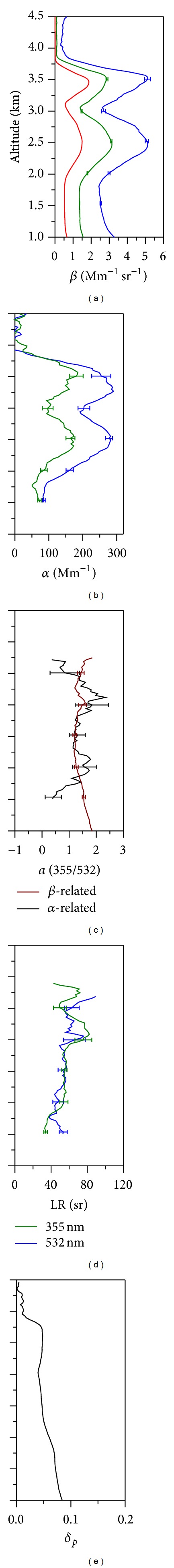
Profiles of (a) backscatter coefficients (355-blue, 532-green, and 1064 nm-red), (b) extinction coefficients (355-blue and 532 nm-green), (c) backscatter and extinction-related Ångström exponent (wavelength pair 355/532 nm), (d) lidar ratios (355 and 532 nm), and (e) particle linear depolarization ratio (532 nm) measured on 18 October 2010 21:45–22:15 UTC.

**Figure 6:**
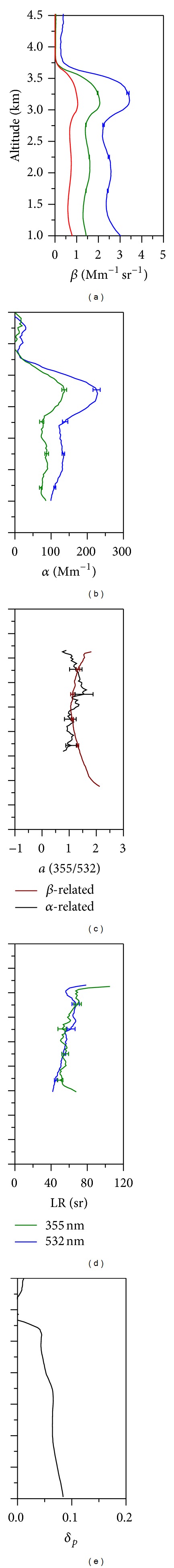
Profiles of (a) backscatter coefficients (355-blue, 532-green, and 1064 nm-red), (b) extinction coefficients (355-blue and 532 nm-green), (c) backscatter and extinction-related Ångström exponent (wavelength pair 355/532 nm), (d) lidar ratios (355 and 532 nm), and (e) particle linear depolarization ratio (532 nm) measured on 19 October 2010 00:00–02:00 UTC.

Figures 3, 4, 5, and 6 show the profiles of the particle backscatter and extinction coefficients, at all available wavelengths, for the different measurement periods, as well as the Ångström exponent, lidar ratio, and particle linear depolarization ratio.

Multilayered structures were visible during the different periods, which can be noticed by the variability within the backscatter and extinction profiles. Maximum values of *β*_355_ above 5 Mm^−1^ sr^−1^ and *α*_355_ close to 300 Mm^−1^ could be observed, namely, during 18 October 2011, while all the most prominent smoke layers detected during the other periods were also characterized by high magnitudes of backscatter and extinction coefficients. The central portions of the most intense layers were visible at heights between 2.5 and 3.5 km and the smoke layer was present at heights up to 4 km during some periods.

**Figure 7:**
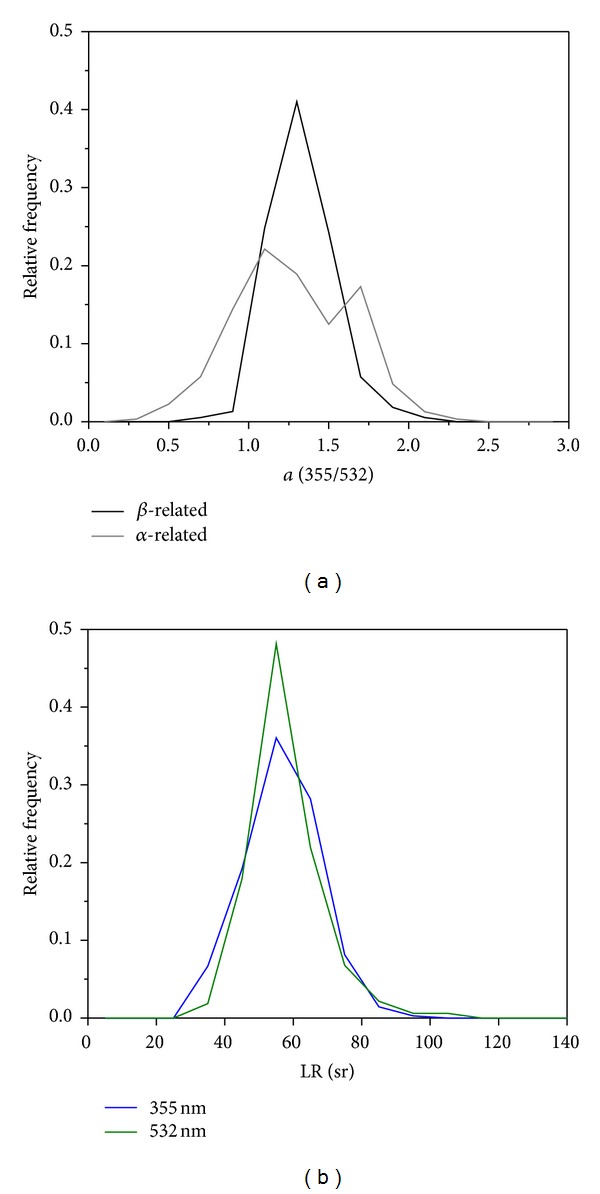
Relative frequency distributions of (a) backscatter and extinction related Ångström exponents (355/532 nm) and (b) lidar ratios at 355 and 532 nm, considering the four different periods of measurements on 17, 18, and 19 October 2011.

The Ångström exponent was usually high, showing a strong wavelength dependency of the backscatter and extinction as indicated in Figures 3–6.  Figure 7 shows the relative frequency of occurrence of å_*β*_ and å_*α*_ for all the data of the four different observational periods (Figures 3–6). Values in the range of 1-2 dominate the distributions. The averaged values of å_*β*_ and å_*α*_ for the layers considered in the inversion algorithm were in the range 1.2–1.6, mainly above 1.4 (Table 1). These magnitudes of the Ångström exponent indicate a predominance of fine mode particles and are comparable to the findings of Alados-Arboledas et al. [27]. They studied a “fresh biomass burning aerosol” plume over Granada and obtained a range of 1.0–1.5 (wavelength pair 355/532 nm) for the backscatter and extinction related Ångström exponents.

**Figure 8:**
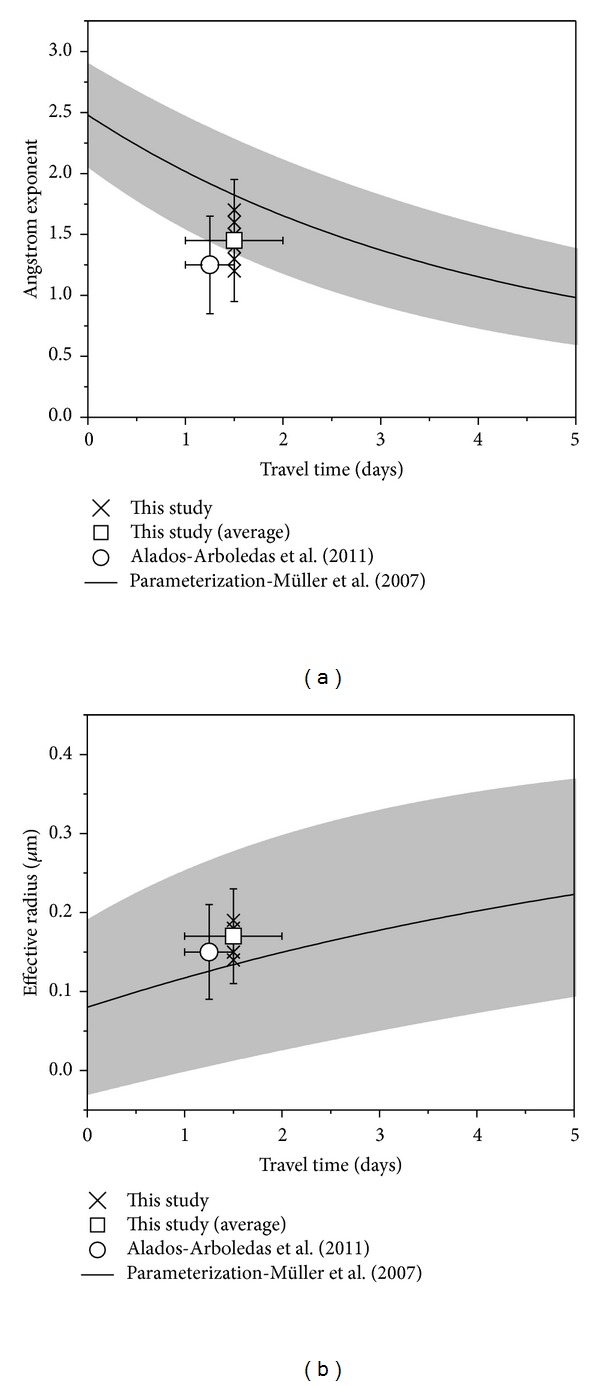
Extinction-related Ångström exponent and effective radius of forest fires smoke, based on Raman lidar measurements versus transport time. The results from the different cases measured at Évora are shown (crosses) as well as the average values (open squares). The average values from Alados-Arboledas et al. [27] are also shown (circles). The thick lines denote the first-order exponential fitting curves and the shaded areas represent the possible ranges for the parameterization according to the fitting parameters and respective fitting errors from Müller et al. [17].

Analysis of the backward trajectories suggested travel times in the order of one to two days, or less, for the smoke plumes observed at Évora, although affected by some uncertainties due to the different source regions contributing to the aerosol load over Évora. In any case, the estimated travel times, in conjunction with the range of observed å, are fairly consistent with the conclusions of Müller et al. [17] on the increase in size of smoke particles with ageing (Figure 8). It was previously noticed that aged smoke, after long range transport (about 1–2.5 weeks), is characterized by lower Ångström exponents, ranging from about 1 down to near zero [24–26]. Very recently, Nicolae et al. [61] reported 1.93 and 1.37 for smoke layers less than one day old, but for 2- and 3-day-old smoke, lower values, close or lower than one, were observed. Thus, our results regarding the Ångström exponents suggest that the smoke plumes described in this work were relatively fresh and our estimate of the smoke age (based on trajectory analysis) is consistent.

The lidar ratio profiles at 355 and 532 nm, shown in Figures 3–6, despite some small differences, are usually relatively similar presenting no clear wavelength dependence. They were found to be mainly in the range of 45–70 sr (Figure 7) and an average value of 56 ± 6 sr was found for both LR_355_ and LR_532_. Within the smoke layers considered for the inversion algorithm, the two lidar ratios were almost equal, as shown in Table 1, except in one case where the difference between the lidar ratios was larger (LR_355_ = 64 ± 2 sr and LR_532_ = 51 ± 2 sr). These results are indicative of the presence of fresh smoke detected at Évora as our measurements are consistent with previous findings in earlier studies: Alados-Arboledas et al. [27] found lidar ratios between 60 and 65 sr. Cattrall et al. [62] compiled a set of mean lidar ratios for biomass burning aerosols (Table  3 and references therein), directly measured by Raman lidars, which were in the range of 50 and 69 sr (at wavelengths between 490 and 550 nm). The frequency distributions reported by Mariano et al. [63] on measurements of biomass burning aerosols are also comparable to the present work. Moreover, lidar ratios from 40 to 100 sr at 355 nm were observed over Eastern Europe by Amiridis et al. [26], with increasing values being correlated with the increasing ageing of the smoke particles. Müller et al. [25] reported mean values of 46 and 53 sr, respectively, at 355 and 532 nm, for aged smoke advected from North America and Siberia to Germany. In the case of smoke from Siberia, observed in Japan after approximately four days of transport, Murayama et al. [64] reported lidar ratios of 40 and 65 at 355 and 532 nm, respectively. The wavelength dependence of the lidar ratios was previously suggested as an indicator of the smoke aging. For long-range transported smoke the ratio of the lidar ratios (LR_355_/LR_532_) is less than 1 [19, 25, 64], whereas Alados-Arboledas et al. [27] obtained a larger value which was “around unity” for fresh biomass burning aerosol. This is also the average value obtained in the present study (1.00 ± 0.03), which corroborates the previous results and gives additional support to the assumption that relatively fresh smoke was observed in the free troposphere over Évora during this period.

The average value of the particle linear depolarization ratio was 5.0 ± 0.6%. In all investigated periods, consistently small particle depolarization ratios were found, mainly in the range of 4–6%. Our results are in agreement with what should be expected for small size particles which were close to sphericity. Comparable values were also reported by other authors [25, 64].

The particle backscatter and extinction coefficients of the most prominent smoke layers for each of the different periods (a total of six cases) were used as input of the inversion algorithm for the retrieval of the microphysical properties. For doing so, the optical data were averaged over height ranges of 400–600 m centered at the altitudes of maximum intensity (i.e., centers of the plumes). The results of the inversion algorithm are discussed in Section 3.2.

### 3.2. Aerosol Microphysical Properties from Lidar Observations

Table 1 contains the layer mean values of optical and microphysical particle properties of the six aerosol layers analyzed in detail.

In this study, effective radii were small, in the range of 0.14–0.19 *μ*m. Alados-Arboledas et al. [27] obtained a comparable range of effective radii, for both lidar based retrievals (0.13–0.17 *μ*m) and sun and star photometer retrievals (0.19-0.20 *μ*m). O'Neill et al. [65] also found effective radii around 0.14 *μ*m based on sunphotometer observations. Larger effective radii were found for long-range transported smoke (e.g., [64, 66]). The parameterization developed by Müller et al. [17], which describes the evolution of particle growth with transport time, seems to be appropriate to the results of this study, similarly to the extinction-related Ångström exponent discussed in the previous section. Figure 8 shows these data, as well as the average of the results reported by Alados-Arboledas et al. [27].

Volume concentrations in the centers of the plumes varied from 18 to 34 *μ*m^3^ cm^−3^, and surface concentration from 376 to 547 *μ*m^2^ cm^−3^.

Real and imaginary parts of the refractive index were derived from the inversion of the lidar data for each case. The real part of the refractive index varied between 1.49 and 1.61, while the imaginary part was in the range 0.01–0.024. Given the comparably large uncertainties, the refractive indexes are not so different between the different cases. The retrievals reported by Alados-Arboledas et al. [27], resulting in a real part of the refractive index between 1.49 and 1.53 and in an imaginary part of 0.02, are comparable to this study. Further consistency can be found with Nicolae et al. [61] (and references therein) which reported a decrease in the imaginary part from fresh (few hours) to aged smoke particles.

The single scattering albedo (SSA) varied between 0.82 and 0.96 with no clear wavelength dependence between 355 and 532 nm. At 1064 nm somewhat smaller values of the single scattering albedo were found, which was also observed by Dubovik et al. [16], whereas in this case Alados-Arboledas et al. [27] reported a “slightly positive spectral dependence.” Regarding the interpretation of the single scattering albedo, one should take into consideration that no spectral dependence on the refractive index is taken into account in the algorithm; thus the spectral dependence of SSA is only driven by the effect of the particle size distribution.

## 4. Summary

Free tropospheric smoke plumes were detected over Évora, Portugal, with Raman lidar during October 2011. Additional ground-based sunphotometer measurements, during daytime, were also used for a basic characterization of the aerosol population present in the column over the site. The main findings of our study can be formulated as follows.

The smoke particles were transported towards Évora from various regions in the Iberian Peninsula, mainly from the north-western areas facing the Atlantic Ocean, as well as from southern Spanish areas, where numerous forest fires were active.

The transportation paths suggested on the presence of relatively fresh smoke, with a lifetime of about 1-2 days, or less, and the optical and physical properties derived from the measurements also indicated that the observed smoke was not aged but relatively fresh.

These aerosol layers were observed up to about 4 km. Particle backscatter coefficients greater than 5 Mm^−1^ sr^−1^ and particle extinction coefficients close to 300 Mm^−1^, at 355 nm, were measured in the center of the layers aloft.

The wavelength dependence of the backscatter and extinction coefficients was usually high, indicating the presence of small particles, which is in agreement with the results obtained from the sunphotometer and MODIS satellite data. Furthermore, in general the lidar ratio presented no clear wavelength dependence and the particle depolarization ratio was consistently low, about 5%.

The microphysical properties retrieved by inversion of the lidar data provided effective radius below 20 *μ*m which is less than values previously observed for aged smoke particles. The real part of refractive index was about 1.5-1.6, while the imaginary part varied around 0.02*i*, indicating fairly strong light absorption characteristics. The single scattering albedo values ranged mainly between 0.85 and 0.93, indicating also the absorption characteristics of the observed particles.

## Figures and Tables

**Table 1:** Mean values of the optical and microphysical properties of the aerosol layers observed between 17 and 19 October 2011.

	Day					
	17 October 2011		18 Oct 2011			19 October 2011
Time (UTC)	20:20–22:10		19:00–20:45	21:45–22:15		00:07–02:05
Layer center, m	2500	3600	2800	2500	3400	3250
Thickness (m)	500	600	600	400	600	500
AOD355	0.09	0.13	0.12	0.11	0.16	0.10
AOD532	0.05	0.07	0.06	0.07	0.09	0.06
LR_355_, sr	52 ± 1	66 ± 3	64 ± 2	55 ± 1	64 ± 10	64 ± 4
LR_532_, sr	49 ± 5	64 ± 7	51 ± 2	55 ± 2	65 ± 9	66 ± 4
LR_532_/LR_355_	0.93 ± 0.11	0.97 ± 0.09	0.81 ± 0.02	0.99 ± 0.03	1.04 ± 0.24	1.04 ± 0.09
å_*α*-355,532_	1.6 ± 0.3	1.5 ± 0.2	1.7 ± 0.1	1.2 ± 0.1	1.4 ± 0.5	1.3 ± 0.1
å_β-355,532_	1.4 ± 0.02	1.5 ± 0.02	1.1 ± 0.02	1.2 ± 0.02	1.4 ± 0.1	1.4 ± 0.1
*δ*_*p*,532_	0.050 ± 0.002	0.038 ± 0.002	0.046 ± 0.003	0.047 ± 0.001	0.046 ± 0.003	0.046 ± 0.003
Reff (*μ*m)	0.15 ± 0.03	0.18 ± 0.04	0.14 ± 0.03	0.19 ± 0.06	0.19 ± 0.06	0.19 ± 0.06
*S* (*μ*m^2^ cm^−3^)	376 ± 130	403 ± 144	487 ± 169	547 ± 171	451 ± 186	382 ± 168
*V* (*μ*m^3^ cm^−3^)	18 ± 8	25 ± 10	23 ± 10	34 ± 14	29 ± 13	25 ± 11
CRI_real_	1.61 ± 0.13	1.49 ± 0.13	1.56 ± 0.14	1.58 ± 0.14	1.52 ± 0.14	1.51 ± 0.13
CRI_imag_	0.018 ± 0.012	0.010 ± 0.010	0.021 ± 0.016	0.024 ± 0.017	0.017 ± 0.014	0.017 ± 0.015
SSA_355_	0.92 ± 0.05	0.96 ± 0.04	0.91 ± 0.06	0.89 ± 0.06	0.93 ± 0.05	0.92 ± 0.06
SSA_532_	0.91 ± 0.05	0.95 ± 0.04	0.90 ± 0.07	0.89 ± 0.07	0.93 ± 0.05	0.92 ± 0.06
SSA_1064_	0.85 ± 0.09	0.92 ± 0.07	0.82 ± 0.12	0.85 ± 0.10	0.89 ± 0.09	0.89 ± 0.09
